# TRPP2 and STIM1 form a microdomain to regulate store-operated Ca^2+^ entry and blood vessel tone

**DOI:** 10.1186/s12964-020-00560-7

**Published:** 2020-08-31

**Authors:** Jizheng Guo, Ren Zhao, Muyao Zhou, Jie Li, Xiaoqiang Yao, Juan Du, Jiexia Chen, Bing Shen

**Affiliations:** 1 School of Basic Medical Sciences, Anhui Medical University, 81 Meishan Road, Hefei, 230032 Anhui China; 2 Department of Cardiology, The First Affiliated Hospital of Anhui Medical University, Hefei, 230032 Anhui China; 3 School of Biomedical Sciences the Chinese University of Hong Kong, Hong Kong, China; 4 Li Ka Shing Institute of Health Science, The Chinese University of Hong Kong, Hong Kong, China; 5 Shenzhen Research Institute, The Chinese University of Hong Kong, Shenzhen, China; 6 Department of Geriatrics Cardiology, The First Affiliated Hospital of Anhui Medical University, 218 Jixi Road, Hefei, 230022 Anhui China; 7 Anhui Province Key Laboratory of Reproductive Health and Genetics, The First Affiliated Hospital of Anhui Medical University, Hefei, 230022 Anhui China

**Keywords:** Polycystin-2, Stromal interaction molecule 1, Vascular smooth muscle cells, Store-operated Ca^2+^ entry, Blood vessel tone

## Abstract

**Background:**

Polycystin-2 (TRPP2) is a Ca^2+^ permeable nonselective cationic channel essential for maintaining physiological function in live cells. Stromal interaction molecule 1 (STIM1) is an important Ca^2+^ sensor in store-operated Ca^2+^ entry (SOCE). Both TRPP2 and STIM1 are expressed in endoplasmic reticular membrane and participate in Ca^2+^ signaling, suggesting a physical interaction and functional synergism.

**Methods:**

We performed co-localization, co-immunoprecipitation, and fluorescence resonance energy transfer assay to identify the interactions of TRPP2 and STIM1 in transfected HEK293 cells and native vascular smooth muscle cells (VSMCs). The function of the TRPP2-STIM1 complex in thapsigargin (TG) or adenosine triphosphate (ATP)-induced SOCE was explored using specific small interfering RNA (siRNA). Further, we created TRPP2 conditional knockout (CKO) mouse to investigate the functional role of TRPP2 in agonist-induced vessel contraction.

**Results:**

TRPP2 and STIM1 form a complex in transfected HEK293 cells and native VSMCs. Genetic manipulations with TRPP2 siRNA, dominant negative TRPP2 or STIM1 siRNA significantly suppressed ATP and TG-induced intracellular Ca^2+^ release and SOCE in HEK293 cells. Inositol triphosphate receptor inhibitor 2-aminoethyl diphenylborinate (2APB) abolished ATP-induced Ca^2+^ release and SOCE in HEK293 cells. In addition, TRPP2 and STIM1 knockdown significantly inhibited ATP- and TG-induced STIM1 puncta formation and SOCE in VSMCs. Importantly, knockdown of TRPP2 and STIM1 or conditional knockout TRPP2 markedly suppressed agonist-induced mouse aorta contraction.

**Conclusions:**

Our data indicate that TRPP2 and STIM1 are physically associated and form a functional complex to regulate agonist-induced intracellular Ca^2+^ mobilization, SOCE and blood vessel tone.

Video abstract

## Background

Polycystin-2 (TRPP2) is a Ca^2+^-permeable channel belonging to the transient receptor potential (TRP) superfamily of cation channels [1]. TRPP2 is encoded by the *PKD2* gene and commonly thought to assemble with the polycystic kidney disease 1 (PKD1) protein to form a receptor-ion channel complex, which is widely expressed in a variety of cell types and tissues including renal epithelium, hepatic bile ducts, pancreatic ducts, vascular smooth muscle cells (VSMCs), and endothelial cells. Mutations in either *PKD2* or *PKD1* gene cause autosomal dominant polycystic kidney disease [2]. At subcellular levels, TRPP2 proteins have been found to be located in the cilia, plasma membrane and endoplasmic reticulum (ER) [3], where they function distinctively by interaction with different partners. In the plasma membrane, TRPP2 may assemble with TRPC1 or TRPV4 to form heteromeric channels participating in mechanosensing and cilium-based Ca^2+^ signaling [4, 5]. In the ER, TRPP2 may associate with inositol 1, 4, 5-triphosphate (IP_3_) receptor (IP_3_R) to serve as an ER Ca^2+^ release channel [6–8]. Functionally, TRPP2 has been shown to regulate cilia movement, apoptosis, mechanosensing, left-right asymmetry, sperm movement and male fertility, and cardiovascular function [9–12].

In many non-excitable and some excitable cells, Ca^2+^ store depletion from the ER activates an influx pathway by which extracellular Ca^2+^ enters the cell to refill the store and carry out specific functions that depend on the “store-operated Ca^2+^ entry” (SOCE) [13]. Accumulating evidence suggests that stromal interaction molecule 1 (STIM1) and Orai1 are key players of SOCE [14]. STIM1 is a single transmembrane protein located in the ER membrane, where its N-terminus protrudes/extends to the ER lumen to function as a Ca^2+^ sensor [15], while its C-terminus is exposed to the cytosol. Depletion of ER Ca^2+^ causes STIM1 to oligomerize to form puncta [16]. The polymerized C-terminus of STIM1 in turn activates Orai1, a plasma membrane protein consists of four transmembrane domain and forms channels to mediate SOCE and the activation of Orai1 allows external Ca^2+^ to enter the cell [17–19]. It has been shown in a number of cell types that while the knockdown of STIM1 with specific siRNA significantly reduced SOCE, the overexpression of STIM1 resulted in a modestly enhanced SOCE [19, 20].

TRPP2 mediates the release of intracellular Ca^2+^ and regulates the influx of extracellular Ca^2+^ to increase intracellular Ca^2+^ concentration [8]. But still, the mechanism of TRPP2 in regulates Ca^2+^ signaling have not yet been fully elucidated. Here we report that TRPP2 is physical associated with STIM1 in the ER membrane in both transfected human embryonic kidney 293 (HEK293) cells and native VSMCs. We thus speculated that TRPP2-STIM1 complex plays distinct roles in SOCE and agonist-induced contraction of VSMCs.

## Methods

### Materials

Phenylephrine (Phe, α receptor agonist), acetylcholine, endothelin 1 (ET-1, endothelin receptor agonist), adenosine 5′-triphosphate disodium salt (ATP-Na_2_), 2-aminoethyl diphenylborinate (2APB, inositol triphosphate receptor inhibitor) were purchased from Sigma and dissolved in the distilled water. Thapsigargin (TG) was obtained from Calbiochem and dissolved in dimethyl sulfoxide (DMSO). The primary goat (sc-10,377) and rabbit (sc-25,749) antibodies against TRPP2, the primary rabbit antibody against IP_3_ receptor and the primary rabbit antibody against Orai1 were purchased from Santa Cruz Biotechnology. The primary rabbit antibody against STIM1 was obtained from ProSci. Fluo-4 fluorescence dye, TRPP2 small interfering RNA (siRNA), STIM1 siRNA, RNAiMax reagent, lipofectamine 2000, goat anti-rabbit IgG conjugated to Alexa Fluor 488 were purchased from Invitrogen. Protein A magnetic bead was obtained from Millipore.

### Cell preparation and culture

All animal experiments were conducted in accordance with NIH publication no. 8523 and were approved by the Animal Experimentation Ethics Committee of Anhui Medical University. Mice were killed by CO_2_ overdose. VSMCs were isolated according to our previous study [21]. Briefly, thoracic aorta was cut out, and the artery lumen was cut open longitudinally. The endothelial layer was mechanically removed by rubbing the lumen with cotton. The smooth muscle tissues were torn out from the adventitial layers, and were then incubated in a Ca^2+^-free phosphate buffer saline (PBS) containing 0.2% collagenase type 1A, 0.9% papain, 0.5% bovine serum albumin (BSA), and 10 mmol/L dithiothreitol at 37 °C for 50 min. VSMCs were dispersed by Pasteur pipette and washed with PBS. VSMCs were cultured for 5–7 days before the experiment. Both VSMCs and HEK293 cell were cultured in dulbecco modified eagle medium (DMEM) supplemented with 10% fetal bovine serum, 100 μg/ml penicillin and 100 U/ml streptomycin.

### Animals preparation

Adult male mice (20 g, 4 weeks old) were housed in a temperature and humidity-controlled vivarium with a 12−/12 h light/dark cycle with access to food and water ad libitum.

### Cloning and transfection

Wild type hTRPP2 (GenBank: U50928.1) and hSTIM1 (GenBank: JX014264.1) were inserted into pEGFP-N1 (at BamH I site), pmCherry-N1 (at BamH I site) or pEGFP-C1 (at Bgl II site) vectors by InFusion Cloning Kit (Clontech Bioinformatics, U.S.). For short N TRPP2, 2-111aa or 112-221aa was deleted from N terminus of TRPP2.

Transfection condition was performed as described previously [22]. HEK293 cells were transfected with all constructs using lipofectamine 2000. About 6 × 10^4^ HEK293 cells were grown in the each well of the 6-well plates. The transfection was performed with 2 μg plasmid and 4 μl lipofectamine 2000 in 200 μl Opti-MEM reduced serum medium in the 6-well plates. The functional studies were performed 3 days post-transfection. Mouse STIM1 and TRPP2 siRNA sequences information were obtained from the literature. The sequence for mouse STIM1-siRNA was UACAGUGGCUCAUUACGUAUU (sense strand) [23]. The sequence for human STIM1-siRNA was AAGGGAAGACCUCAAUUACCA (sense strand) [24]. The TRPP2-siRNA sequence was AACCUGUUCUGUGUGGUCAGGUUAU (sense strand), and was used in both human and mouse species [25]. Scrambled siRNA sequence is UAACGACGCGACGACGUAA (sense strand). Small interfering RNA delivery was achieved by lipofectamine RNAiMAX reagent according to the manufacturer manual. In the vessel tissue, siRNA was applied into culture medium overnight with lipofectamine RNAiMAX. The blood vessels were used in the experiment, after 24 h culture.

### Fluorescence resonance energy transfer (FRET)

Sensitized emission FRET was performed as described previously [26]. According to Leica confocal software manual, GFP is a donor fluorophore in the GFP-mCherry FRET pair, while mCherry is an acceptor fluorophore to accept GFP emission. TRPP2 and STIM1 were tagged with GFP and mCherry, respectively. Donor only cells transfected with the GFP-tagged construct and acceptor only cells transfected with the mCherry-tagged construct were utilized as references. The references are used to obtain calibration coefficients to correct for excitation and emission cross talk. According to the routine of FRET workflow, FRET efficiency was collected in HEK293 cells co-transfected with GFP-tagged and mCherry-tagged constructs and calculated by following equation [27]:

$$ {E}_{\mathrm{FRET}}=\left(\mathrm{B}\hbox{-} \mathrm{A}\times \upbeta \hbox{-} \mathrm{C}\times \upgamma \right)/\mathrm{C} $$

In the equation, A, B and C are donor, FRET and acceptor channel intensities respectively. As the calibration factors, β is the FRET channel intensity/donor channel intensity in the donor only cells and γ is the FRET channel intensity/acceptor channel intensity in the acceptor only cells.

### Intracellular calcium ([Ca^2+^]_i_) measurement

[Ca^2+^]_i_ was measured according to our previous report [21]. Briefly, the cells were loaded with 10 μmol/L Fluo-8/AM and 0.02% pluronic F-127 dissolved in a normal physiological saline solution (NPSS) that contained in mmol/L: 140 NaCl, 5 KCl, 1 CaCl_2_, 1 MgCl_2_, 10 glucose, 5 Hepes, pH 7.4 at 37 °C for 1 h in dark. The cell Ca^2+^ store was depleted by 10 μmol/L ATP or 2 μmol/L thapsigargin in the Ca^2+^-free solution containing in mmol/L: 140 NaCl, 5 KCl, 1 MgCl_2_, 10 glucose, 0.2 ethylene glycol-bis(β-aminoethyl ether)-N,N,N′,N′-tetraacetic acid (EGTA), 5 Hepes, pH 7.4. Ca^2+^ influx was initiated by applying 1 mmol/L Ca^2+^ in bath solution. The fluorescence signal was recorded and analyzed by TCS SP5 confocal laser scanning system (Leica, Germany). Changes in the peak value of cytosolic [Ca^2+^]_i_ were displayed as a ratio of fluorescence relative to the baseline intensity before the application of ATP/TG or extracellular Ca^2+^ (F_1_/F_0_).

### Co-immunoprecipitation and immunoblots

Co-immunoprecipitation and immunoblots were performed according to our previous report [21]. The proteins were extracted from the aorta or cells with detergent extraction buffer containing 1% Nonidet P-40, 150 mmol/L NaCl, and 20 mmol/L Tris-HCl, pH 8.0, plus protease inhibitor cocktail tablets. TRPP2 or STIM1 proteins were immunoprecipitated by incubating 800 μg of the extracted proteins with 7 μg of anti-TRPP2 or anti-STIM1 antibody on a rocking platform overnight at 4 °C. Protein A agarose was then added and incubated for another 3 h at 4 °C. The immunoprecipitates were washed with the lysis buffer for 3 times and were resolved on an SDS/PAGE gel. For the immunoblots, the poly (vinylidene difluoride) membrane carrying transferred proteins was incubated at 4 °C overnight with respective primary antibodies: anti-STIM1, anti-TRPP2, anti-IP_3_R, anti-Orai1 and β-tubulin (1:200). Immunodetection was accomplished using horseradish peroxidase-conjugated secondary antibody and ECL detection system. The optical density of each blot was normalized to that of β-tubulin and expressed as the relative optical density.

### Proximity ligation assay (PLA)

In situ PLA kit Duolink (Sigma-Aldrich, U.S.) was used to detect the interaction of TRPP2 and STIM1 according to the manufacturer’s instructions and previous study [28]. Briefly, fresh isolated VSMCs from mesenteric arteries were fixed and permeabilized. Next, VSMCs were blocked with Duolink blocking solution and incubated with anti-TRPP2 (Santa Cruz, sc-10,377, U.S.) [29] and anti-STIM1 (Santa Cruz, sc-68,897, U.S.) [30] (1:40, each) antibodies overnight at 4 °C in Duolink antibody diluent. Negative control cells were incubated with anti-STIM1 antibody alone. After the following washout with physiological saline solution, the VSMCs were incubated with Duolink secondary antibodies conjugated with oligonucleotides (anti-goat PLA probe Plus (Sigma, DUO92003, U.S.) and anti-rabbit PLA probe Minus (Sigma, DUO92005, U.S.) in a pre-heated humidity chamber for 1 h at 37 °C. Then the VSMCs were incubated with a ligation solution containing two oligonucleotides and one ligase. When two proteins were in close proximity (< 40 nm separation), the oligonucleotides would hybridize to the two PLA probes. Subsequently, the ligase would join the two hybridized oligonucleotides to form a close circle. A rolling-circle amplification reaction using the ligated circle as a template would result in a repeated sequence product. A fluorescence (Texas Red channel)-labeled complementary oligonucleotide detection probes (Sigma, DUO92008, U.S.) were used to detect the amplification products. The VSMCs were mounted with a medium containing 4′,6-diamidino-2-phenylindole (DAPI) nuclear stain. The fluorescence signals (positive signals: red fluorescent dots) were visualized and imaged using a TCS SP5 confocal microscope (Leica, Germany).

### Immunofluorescence

Immunofluorescence was performed as described elsewhere [31]. Cultured aortae or primary cultured VSMCs were fixed with 4% formaldehyde for overnight or 10 min respectively, followed by permeabilization with 0.1% Triton X-100 dissolved in PBS. The samples were blocked by 2% BSA at room temperature for 1 h before incubating with primary antibody at 4 °C overnight. After washing with PBS for three times, the samples were incubated with donkey anti-rabbit IgG conjugated to Alexa Fluor 488 (1:200) for 1 h at room temperature and mounted in 90% glycerol in PBS and the fluorescent signals were determined by a TCS SP5 confocal laser system (Leica, Germany). The 8-bit images were analyzed with ImageJ software [32]. Briefly, projected images were generated by collecting maximum pixel intensity of the in-focus frames into a single frame. The images were threshold to remove background fluorescence and used to create a mask image. Using the mask images, number of STIM1 puncta was scored with automatic “Analyze Particles” algorithm of ImageJ software and using cluster size of 3–100 pixels and circularity 0.1–1.0.

### Generation of *PKD2* (TRPP2, smooth muscle) conditional knockout (CKO) mice

*Pkd2*^*cond*^ mutant mice purchased from Jackson Laboratory (Stock number: 017292; B6.129X1(Cg)-*Pkd2*^*tm1.1Tjwt*^/J, U.S.) possess *loxP* sites flanking exons 11–13 of *Pkd2* gene [33]. In the development of the *Pkd2*^*cond*^ mutant mice, a targeting vector was constructed. A *loxP* site was inserted on the upstream of exon 11 and a second *loxP* site, followed by a *frt*-flanked neomycin resistance (neo) cassette was inserted on downstream of exon 13 of *Pkd2* gene. The *Pkd2* targeting vector was electroporated into 129X1/SvJ-derived embryonic stem (ES) cells. ES cells were injected into blastocysts. Chimeric mice were obtained by breeding to C57BL/6 J mice. To delete the neo cassette, *Flp* transgenic mice were used to bred with offspring. Next, the resulting homozygous for the floxed-Pkd2 (*Pkd2*^*cond*^) allele were obtained after the progeny were crossed to remove the *Flp*-expressing transgene. Floxed *Pkd2* mice were then crossed with STOCK Tg(Tagln-cre)1Her/JNju mice bearing a Cre-recombinase (Fig. 8a). DNA isolated from mice tail tissues of the offspring were genotyped by PCR for *LoxP*^+/+^ sites and the presence of the Cre-recombinase using specific primers (For *Pkd2 LoxP*^+/+^, forward primer sequence: 5′-GGGGTTCCTATGAAGAGTTCCAAG-3′, the revers primer sequence: 5′-CTGACAGGCACCTACAGAACAGTG-3′; For Cre, the forward primer sequence: 5′-ATTTGCCTGCATTACCGGTC-3′, the reverse primer sequence: 5′-ATCAACGTTTTCTTTTCGG-3′). A representative genotyping result showed *Lox P* (+/+) sites (485 bp), Cre control (350 bp) and wild type (382 bp) (Fig. 8b). TRPP2 expressing in mice aortic smooth muscle was confirmed by immunoblots (Fig. 8c). *PKD2*^+/+^-Cre was as Cre control. All breeding and animal studies were approved by the Animal Experimentation Ethics Committee of Anhui Medical University.

### Vessel tension measurement

Vessel tension measurement was performed as described in our previous report [34]. Briefly, after euthanasia, the mouse thoracic aorta was quickly dissected free and placed in Krebs Henseleitt solution consisting of (in mmol/L): NaCl 118, KCl 4.7, CaCl_2_ 2.5, KH_2_PO_4_ 1.2, MgSO_4_ (7 H_2_O) 1.2, NaHCO_3_ 25.2 and glucose 11.1. Under a dissecting microscope, adhering perivascular tissue was cut into 2 mm-long rings. The endothelial layer was mechanically removed by gently rubbing the luminal surface. The vessel rings were mounted onto two thin stainless steel holders, one of which was connected to a force displacement transducer and the other to a movable device that allowed the application of passive tension of 0.5 g, a range that was determined to be the optimal resting tension for obtaining maximal active tension induced by a 60 mmol/L K^+^ solution. The mounted rings were kept in 5 ml organ baths containing Krebs Henseleit solution at 37 °C and continuously bubbled with a gas mixture of 95% O_2_ and 5% CO_2_ to maintain a pH of 7.4. The isometric tension was recorded and analyzed by a DMT myograph (model 610 M; Danish Myo Technology, Aarhus, Denmark). After an equilibration period of 60 min, the contractile function of the vessel was tested by replacing Krebs Henseleit solution with 60 mmol/L K^+^ solution that was prepared by replacing NaCl with an equimolar amount of KCl, which was taken as the reference contraction. After the restoration of vessel tension to the baseline levels, the rings were exposed to 10 μmol/L Phe to test their contractile responses, and subsequently challenged with acetylcholine to certify endothelial functional removal. The contractile response to Phe (10^–8.5^-10^− 5.5^ mol/L) or ET-1 (10^− 9^–10^− 7^ mol/L) was obtained by cumulatively adding agonists into the bath with or without the pretreatment of inhibitors for 10 min. The vessel contraction was normalized by vessel weight (contraction/weight (g/g)) to remove the effect of vessel thickness and length on maximal intensity of contraction [35]. Some vessel tissues were loaded with heparin (1 mg/ml) using a reversible permeabilization loading procedure. The tissues were exposed to a series of high K^+^, low Ca^2+^, EGTA containing solutions [36].

### Statistics

Data were expressed as mean ± SEM. The statistical significance was determined using two-tailed Mann-Whitney U test or two-way analysis of variance followed by Games-Howell post hoc tests when more than two treatments were compared. Differences were considered significant with a value of *P* < 0.05. In the [Ca^2+^]_i_ measurements, *n* represents the number of experiments.

## Results

### Physical interaction between heterologously expressed TRPP2 and STIM1 in HEK293 cells

**Fig. 1:**
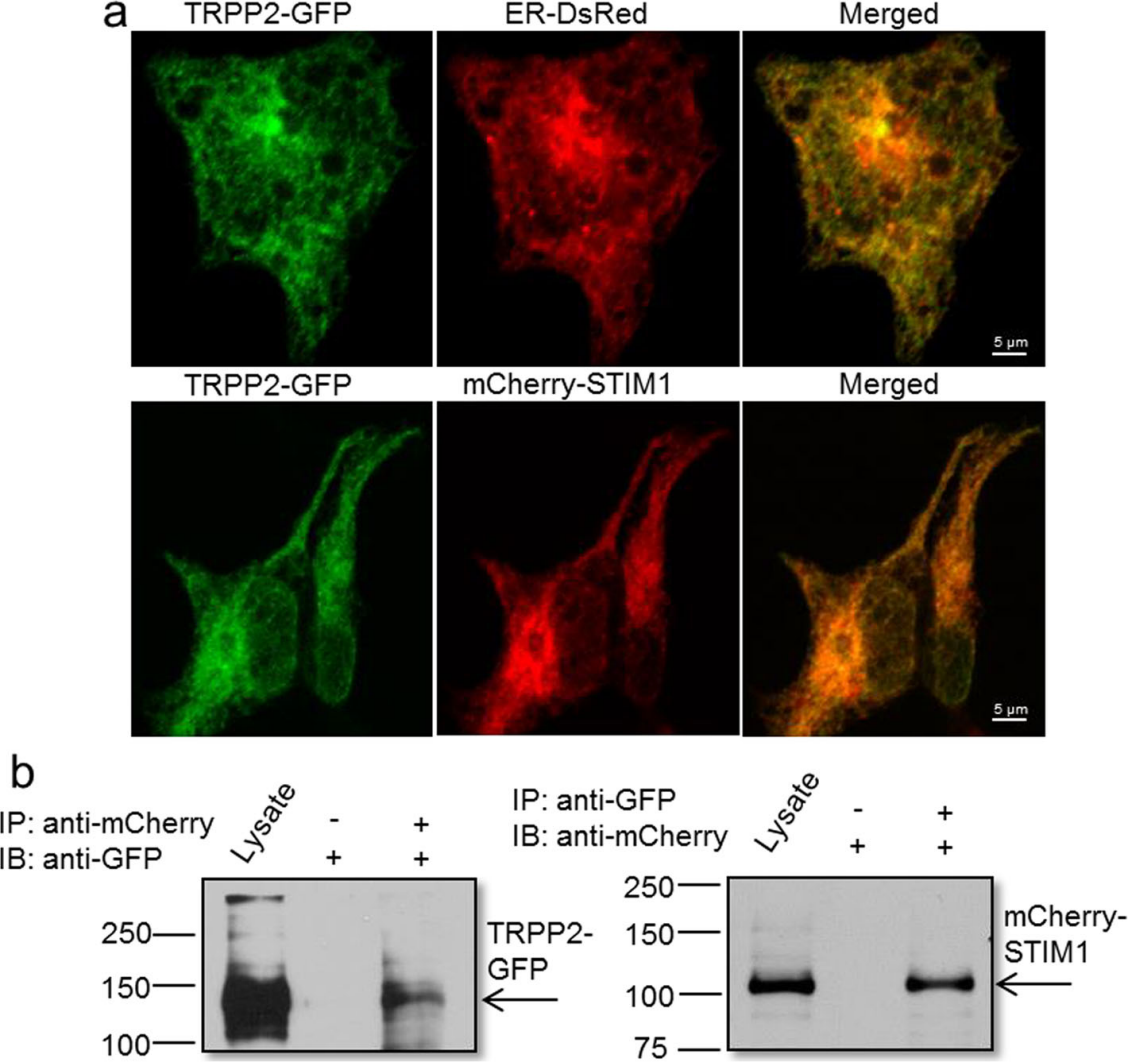
Co-localization and co-immunoprecipitation of TRPP2 and STIM1 in transfected HEK293 cells. **a** Upper penal: GFP-tagged TRPP2 (TRPP2-GFP) co-localized with endoplasmic reticulum maker (ER-DsRed); Lower penal: TRPP2-GFP co-localized with mCherry-tagged STIM1 (mCherry-STIM1). **b** Representative images showing co-immunoprecipitation followed by immunoblots [left, immunoblot with anti-GFP; right, immunoblot with anti-mCherry]. GFP or mCherry antibody pulled down the proteins from TRPP2-GFP and mCherry-STIM1 co-expressing HEK293 cells. The experiment was repeated 4 times

Both TRPP2 and STIM1 are expressed in the ER membrane. To test if TRPP2 interacts with STIM1, C-terminal GFP-tagged TRPP2 (TRPP2-GFP) was co-expressed with N-terminal mCherry-tagged STIM1 (mCherry-STIM1) in HEK293 cells. Double fluorescent imaging showed that TRPP2-GFP colocalized very well with mCherry-STIM1 and ER-DsRed (an ER marker) in the transfected cells (Fig. 1a). Moreover, reciprocal co-immunoprecipitation assays further demonstrated that TRPP2 interacted with STIM1 in the transfected cells (Fig. 1b).

**Fig. 2:**
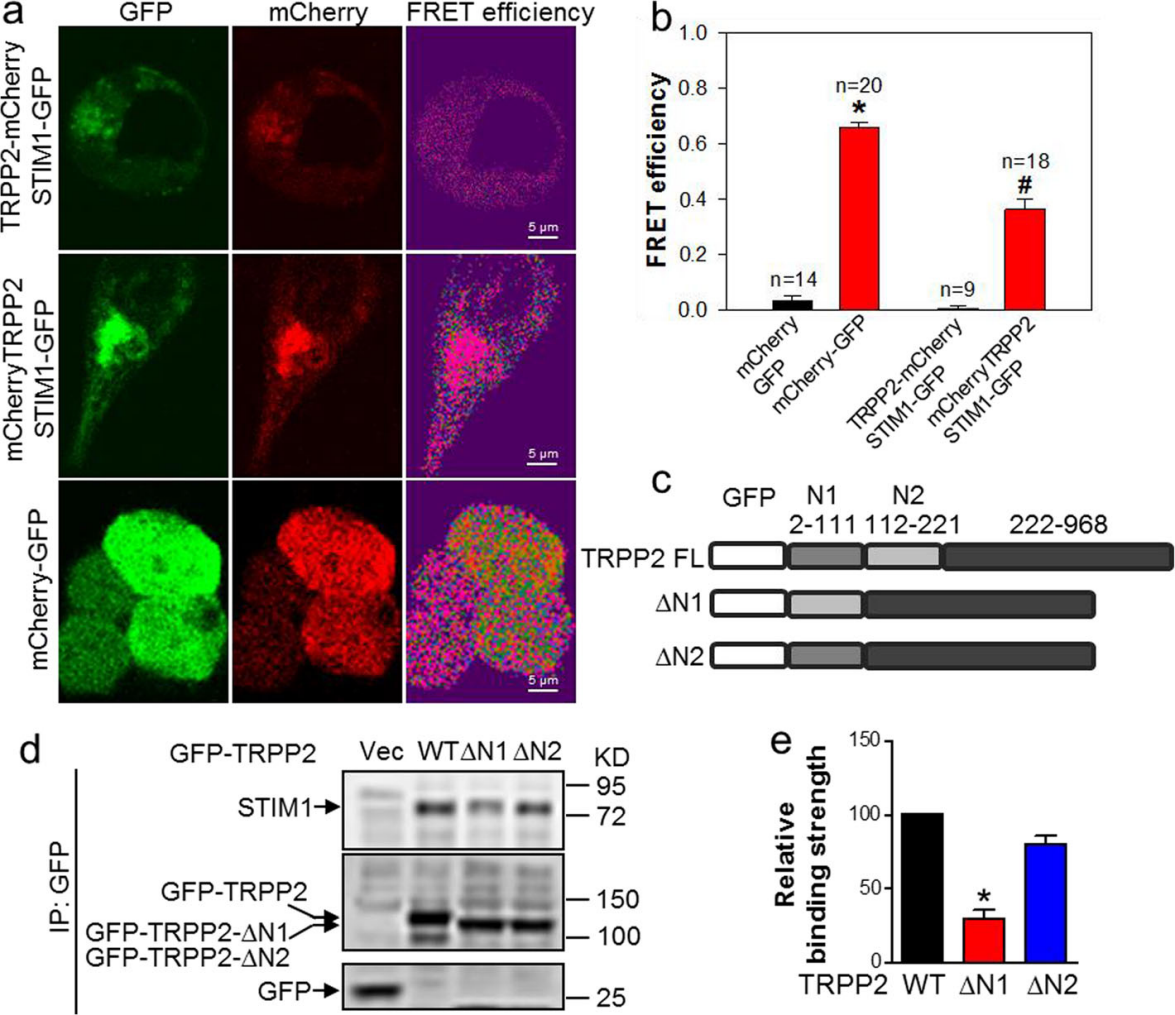
FRET efficiency of TRPP2-STIM1 interaction in TRPP2 and STIM1 co-expressing HEK293 cells. **a** Representative images showing GFP, mCherry and FRET efficiency channels. The cells expressed TRPP2-mCherry and STIM1-GFP (upper), mCherry-TRPP2 and STIM1-GFP (middle) or GFP-mCherry (lower) proteins respectively. **b** Summarized data showing FRET efficiency in transfected HEK293 cells (four groups: mCherry and GFP, mCherry-GFP, TRPP2-mCherry and STIM1-GFP, mCherry-TRPP2 and STIM1-GFP). Values are shown as the mean ± SEM (*n* = 9–20 cells). ^*^P < 0.05 for GFP and mCherry co-expression vs. GFP-mCherry expression; ^#^*P* < 0.05 for TRPP2-mCherry and STIM1-GFP co-expression vs. mCherry-TRPP2 and STIM1-GFP co-expression. **c** Schematic diagram of full-length TRPP2 and its truncated derivatives: TRPP2 without N1 (2-111aa, ∆N1) and TRPP2 without N2 (112-221aa, ∆N2). **d** Representative images of co-immunoprecipitation experiments in HEK293 cells co-expressed with STIM1 plus GFP-tagged full-length TRPP2 (WT) or ∆N1 or ∆N2. IP, GFP antibody; IB, anti-EGFP antibody. The GFP-only vector was used as a negative control. **e** Summarized data showing the relative binding strength of STIM1 with TRPP2 (WT), or ∆N1 or ∆N2. The optical density of ∆N1 or ∆N2 blot was normalized to that of WT blot (= 100%) and expressed as the relative binding strength. Values are shown as the mean ± SEM (*n* = 3). ^*^P < 0.05 for STIM1 and ∆N1 or ∆N2 co-expression vs. STIM1 and TRPP2 co-expression

Next, we used sensitized emission FRET, a powerful tool to identify protein-protein interactions [26], to probe if TRPP2 and STIM1 directly interact with each other. Because both the N- and C-termini of TRPP2 as well as the C-terminus of STIM1 are all located at the cytoplasmic side of the ER membrane [14, 37], we reasoned that if the two proteins interact, one of the cytoplasmic termini of TRPP2 should be in close proximity with the STIM1 C-terminus. To test this, mCherry-tagged TRPP2 with mCherry fused either at the N- or the C-terminus of TRPP2 (mCherry-TRPP2 and TRPP2-mCherry) were individually co-expressed with STIM1-GFP in HEK293 cells. Cells that expressed GFP-mCherry fusion protein were used as positive control, whereas those that co-expressed GFP and mCherry as separate proteins were used as negative control for the FRET experiments. Remarkably, the co-expression of mCherry-TRPP2 and STIM1-GFP led to a high FRET efficiency, to approximately a half of that achieved by the GFP-mCherry fusion protein. By contrast, the co-expression of TRPP2-mCherry and STIM1-GFP produced a very low FRET efficiency as that of GFP and mCherry (Fig. 2a, b). These results suggest that the N-terminus, but not C-terminus, of TRPP2 is closely associated with STIM1 C-terminus. To map the TRPP2 region mediating association with STIM1, we linked each of a series of TRPP2 N-terminus truncation derivatives with GFP tag (Fig. 2c) for Co-IP assay. As showing in Fig. 2d-e, full-length TRPP2 (WT) and its delete N2 (112-221aa, ∆N2) truncated but not delete N1 (2-111aa, ∆N1) truncated derivatives bound strongly to STIM1, suggesting that the N1 domain of TRPP2 is responsible for the interaction.

### Role of TRPP2-STIM1 complex in SOCE

**Fig. 3:**
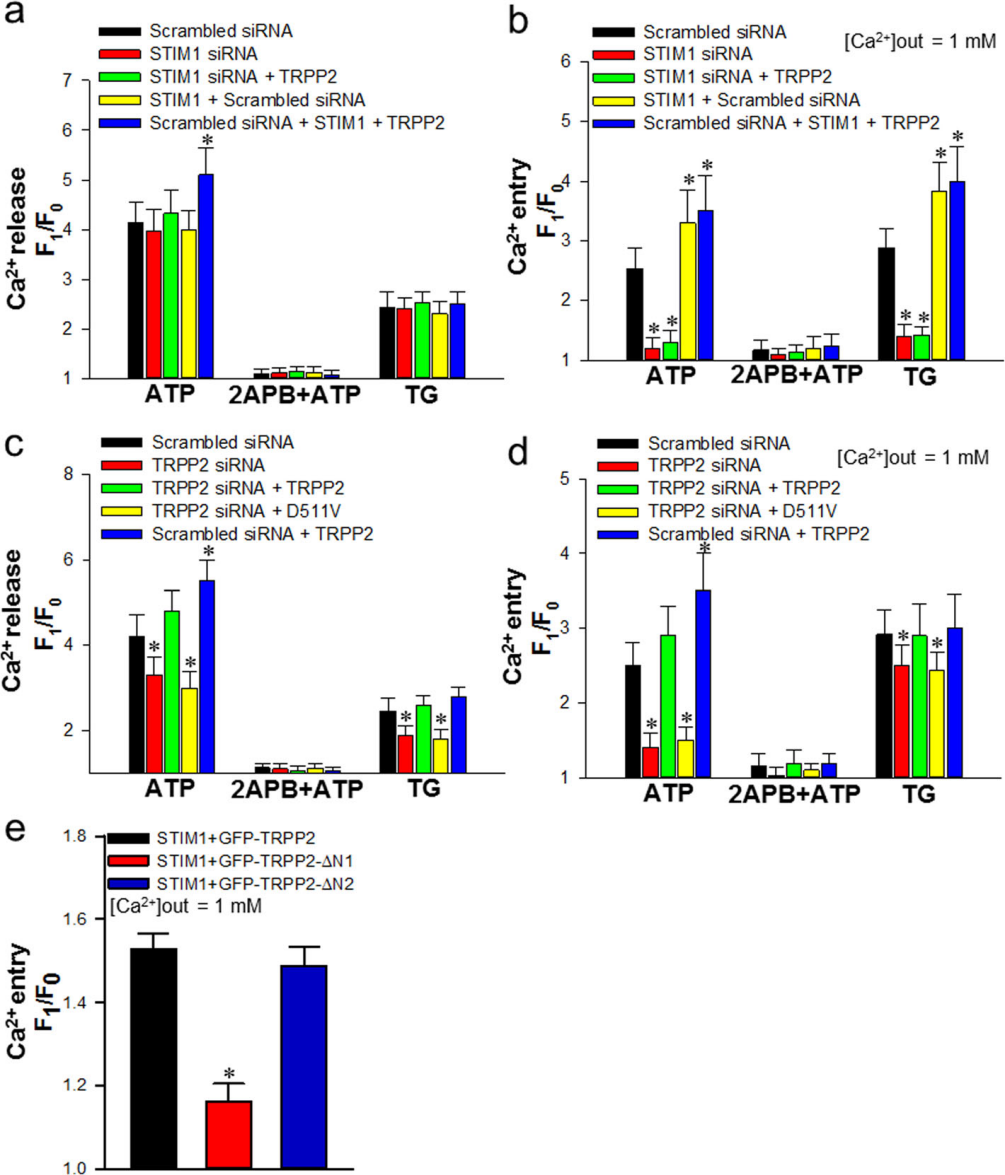
Effect of TRPP2-STIM1 interaction on store-operated Ca^2+^ entry (SOCE) in TRPP2 and STIM1 co-expressing HEK293 cells. **a**-**d** Summary of data showing changes in Ca^2+^ release (**a**, **c**) and SOCE (**b**, **d**) in HEK293 cells transfected with TRPP2 siRNA, STIM1 siRNA, STIM1, TRPP2 and/or dominant negative TRPP2 (D511V), and treated by ATP (10 μmol/L), 2APB (100 μmol/L) + ATP (10 μmol/L) and thapsigargin (TG, 2.5 μmol/L) for 8 min in Ca^2+^-free solution. SOCE was evoked by extracellular Ca^2+^ (1 mmol/L) application. Values are shown as the mean ± SEM (*n* = 3–5). *P < 0.05 compared with scrambled siRNA in each treatment. **e** Summarized data showing ATP (10 μmol/L)-induced SOCE in HEK293 cells co-expressed with STIM1 and GFP-tagged full-length TRPP2 (GFP-TRPP2) or TRPP2 without N1 (GFP-TRPP2-∆N1, deletion of 2-111aa in TRPP2) or TRPP2 without N2 (GFP-TRPP2-∆N2, deletion of 112-221aa in TRPP2). Values are shown as mean ± SEM (*n* = 5). ^*^P < 0.05 for STIM1 and GFP-TRPP2 or GFP-TRPP2-∆N2 co-expression vs. STIM1 and GFP-TRPP2-∆N1 co-expression

It is well established that STIM1 senses ER Ca^2+^ store depletion to activate SOCE [14]. Since TRPP2 interacts with STIM1, it may also play a role in SOCE. To test this hypothesis, we first used siRNA to knock down TRPP2 expression in HEK293 cells. As a control, we also used siRNA for STIM1. The TRPP2 and STIM1 specific siRNAs markedly suppressed the expression of endogenous TRPP2 and STIM1, respectively, in HEK293 cells (Supplementary Fig. 1). We then examined SOCE in the siRNA-transfected cells. Fluo-8 AM loaded cells were treated with TG (2.5 μmol/L), an inhibitor of sarco/endoplasmic reticulum Ca^2+^-ATPase (SERCA) that induces passive ER Ca^2+^ store depletion (Ca^2+^ release) via both IP_3_R-dependent and independent pathways [38], in the absence of extracellular Ca^2+^ for 8 min and then Ca^2+^ (1 mmol/L) was reintroduced to the bath solution. Consistent with the specific role for STIM1 in Ca^2+^ entry, STIM1 siRNA strongly suppressed the increase in [Ca^2+^]_i_ upon reintroduction of extracellular Ca^2+^ (Ca^2+^ entry) (Fig. 3b) but did not alter the TG-evoked [Ca^2+^]_i_ rise in the absence of extracellular Ca^2+^ (Ca^2+^ release) (Fig. 3a). On the other hand, the overexpression of STIM1 moderately increased the TG-evoked Ca^2+^ entry without affecting Ca^2+^ release (Fig. 3a, b). The transfection of TRPP2 siRNA, however, caused significant decreases in both the Ca^2+^ release and Ca^2+^ entry induced by TG (Fig. 3c, d), with the effect on Ca^2+^ entry far less pronounced than that caused by STIM1 siRNA. The inhibitory effects of TRPP2 siRNA on Ca^2+^ release and entry were both rescued by co-expression of wild type TRPP2, but not its dominant negative mutant, D511V [39] (Fig. 3c, d). The overexpression of TRPP2, either in the absence or presence of STIM1 (endogenous or exogenous), did not alter the TG-induced Ca^2+^ release or Ca^2+^ entry (Fig. 3a-d). These results indicate that unlike STIM1, TRPP2 mainly exerts effect on Ca^2+^ release from the ER store, which then affects SOCE.

Studies from other groups have shown that ATP activates TRPP2 indirectly via IP_3_-induced Ca^2+^ release [8]. Thus, ATP (10 μmol/L) was used in place of TG to induce SOCE. Remarkably, the transfection of TRPP2 siRNA significantly suppressed ATP-induced Ca^2+^ release and the Ca^2+^ entry was diminished to a similar degree as that caused by STIM1 siRNA, although the latter exhibited no effect on ATP-induced Ca^2+^ release (Fig. 3a-d). Again, the co-expression of wild type TRPP2, but not the dominant negative D511V mutant, rescued the suppressed ATP-induced Ca^2+^ release and entry in the TRPP2 siRNA-transfected cells (Fig. 3c, d). The overexpression of TRPP2 in the absences of TRPP2 siRNA even enhanced the ATP-induced Ca^2+^ release and the subsequent Ca^2+^ entry (Fig. 3c, d). The enhancing effect of TRPP2 overexpression on ATP-induced Ca^2+^ release was evident even with the overexpression of STIM1 (Fig. 3a). However, for Ca^2+^ entry, the TRPP2-mediated increase was not obvious because STIM1 alone already had a similar effect (Fig. 3b).

Supporting the role of IP_3_Rs in ATP-induced Ca^2+^ release, IP_3_R inhibitor, 2APB (100 μmol/L), abolished the ATP-induced Ca^2+^ release and Ca^2+^ entry under all conditions tested (Fig. 3a-d). Although 2APB may also inhibit SOCE and/or other nonspecific targets [40], the lack of any ATP-induced Ca^2+^ release in the presence and absence of TRPP2 is consistent with the idea that TRPP2 activation in the ER is triggered by IP_3_R-mediated Ca^2+^ release [8].

Next, we sought to suppress the TRPP2-STIM1 interaction in the ER. In above study, we have proved that TRPP2 ∆N1 has a weak interaction with STIM1. Therefore, we used TRPP2 ∆N1 as a dominant negative mutant for TRPP2-STIM1 interaction to identify the functional role of TRPP2-STIM1 complex in the SOCE using live Ca^2+^ fluorescence measurement. The results of [Ca^2+^]_i_ measurement suggested that SOCE of TRPP2 ∆N1 and STIM1 co-transfected cells was remarkably reduced compared to TRPP2 ∆N2 and STIM1 or whole TRPP2 and STIM1 co-transfected HEK293 cells (Fig. 3e).

These results indicate that TRPP2 associates with STIM1 to crucially regulate SOCE process and TRPP2-STIM1 complex has a pivotal role in IP_3_-mediated Ca^2+^ signaling.

### Functional role of TRPP2-STIM1 complex in SOCE in VSMCs

**Fig. 4:**
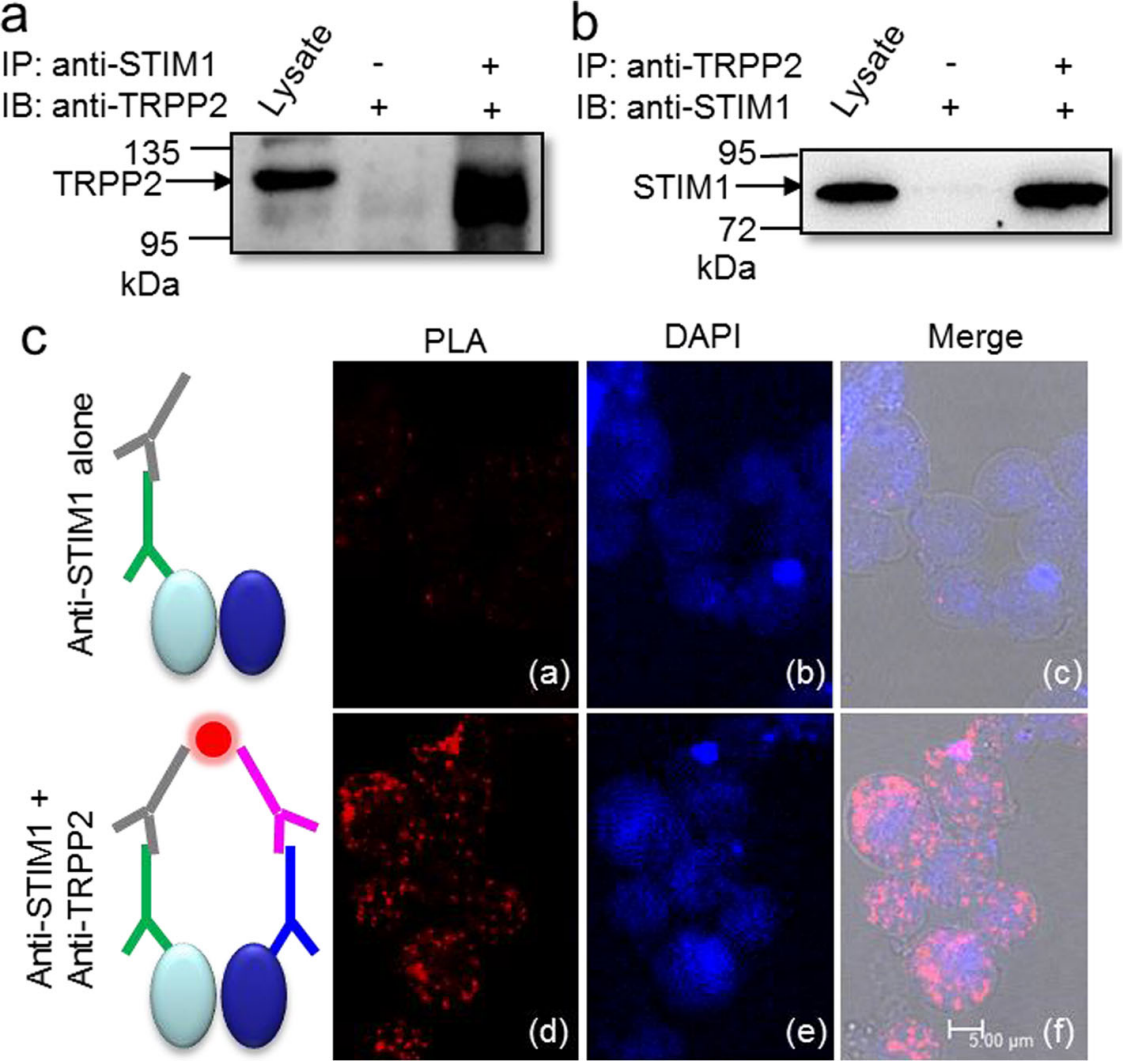
Co-immunoprecipitation and in situ proximity ligation assay (PLA) of TRPP2 and STIM1 in mouse aortic smooth muscle cells. (**a**, **b**) Immunoblots showing that anti-TRPP2 and anti-STIM1 recognized TRPP2 and STIM1 proteins, and co-immunoprecipitation followed by immunoblots (**a**, immunoblot with anti-TRPP2; **b**, immunoblot with anti-STIM1). Proteins from the mouse aortic smooth muscle cells were immunoprecipitated with indicated antibody (+) or no antibody (−). (**c**) PLA analysis was used to detect the interaction between TRPP2 and STIM1. Representative images were displayed in the presence of anti-STIM1 antibody alone ((a)-(c)), or in the presence of anti-TRPP2 and anti-STIM1 antibodies ((d)-(f)). ((c), (f)) were merged with bright view. Red doted fluorescence showing positive signal. Nuclei were marked by DAPI staining (blue color). Scale bar represents 5 μm. The experiment was repeated 4 times

Both TRPP2 and STIM1 are expressed in VSMCs [41, 42]. Hypothesizing that endogenous TRPP2 physically interacts with STIM1 in VSMCs. Reciprocal co-immunoprecipitation assay showed that endogenous TRPP2 and STIM1 pulled down each other in the mouse aortic VSMCs (Fig. 4a, b). Additionally, to confirm the co-localization of TRPP2 and STIM1, we utilized a powerful method PLA, which detects proteins located within a radius of < 40 nm. In the presence of both anti-TRPP2 and anti-STIM1 antibodies, red fluorescent dots indicated a positive signal of PLA in fixed fresh isolated VSMCs (Fig. 4c: (d)-(f)). A negative control incubating with anti-STIM1 antibody alone displayed a negligible number of fluorescent dots (Fig. 4c: (a)-(c)). These results suggest that TRPP2 indeed very colocalizes with STIM1 in mouse aortic VSMCs.

**Fig. 5:**
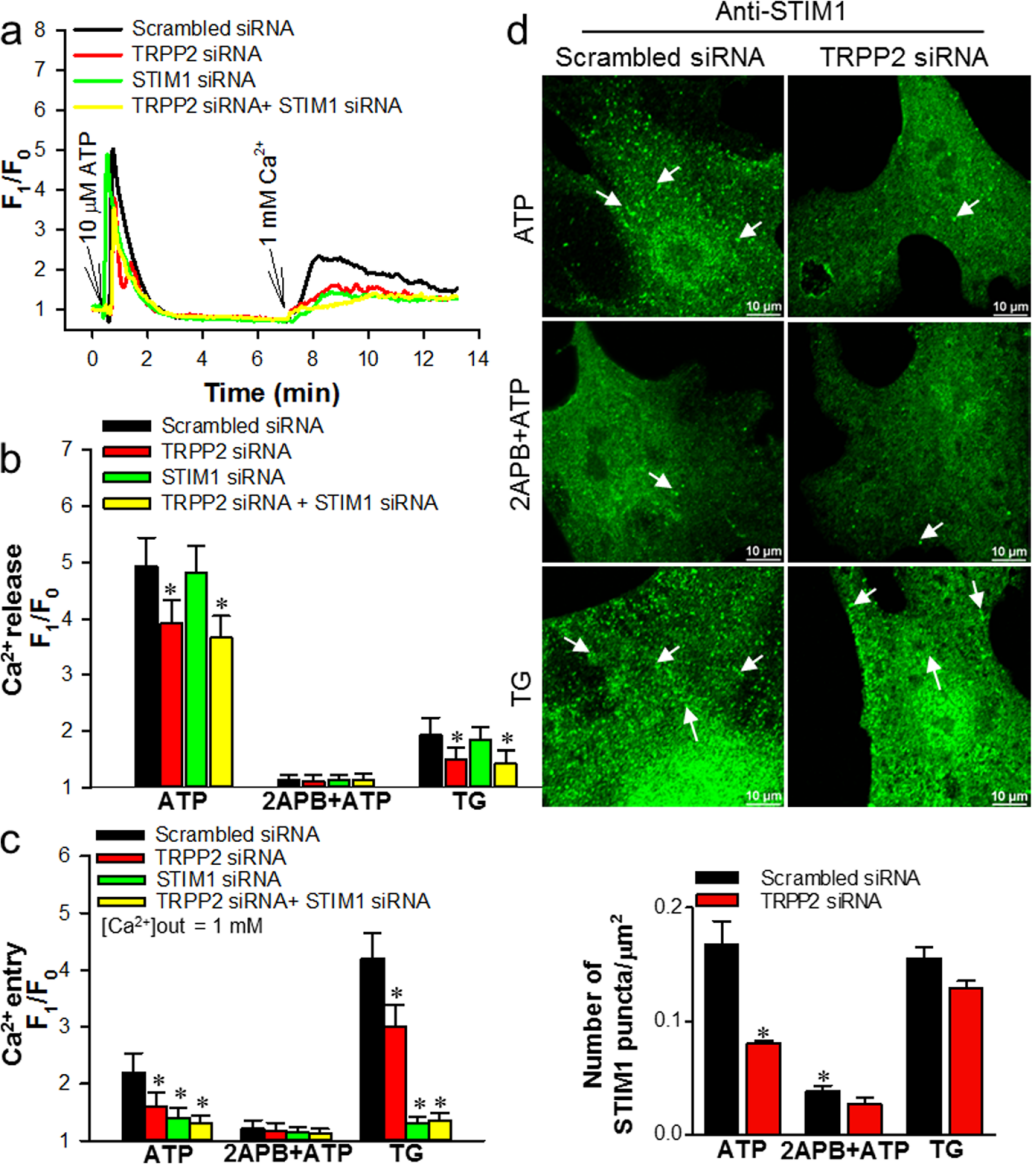
Role of TRPP2 in store-operated Ca^2+^ entry (SOCE) and STIM1 puncta formation in mouse aortic smooth muscle cells. **a** Representative traces showing ATP (10 μmol/L)-induced Ca^2+^ release in Ca^2+^-free solution and SOCE in the mouse aortic smooth muscle cells transfected with TRPP2, STIM1, both TRPP2 and STIM1, or scrambled siRNAs. **b** Summary of data showing changes in intracellular Ca^2+^ concentration increase in response to extracellular ATP (10 μmol/L) or thapsigargin (TG, 2.5 μmol/L) application in the mouse aortic smooth muscle cells treated with or without 2APB (100 μmol/L) for 10 min in Ca^2+^-free solution. **c** Summary of data showing changes in intracellular Ca^2+^ concentration increase in response to extracellular Ca^2+^ (1 mmol/L) application in the mouse aortic smooth muscle cells treated by ATP (10 μmol/L), 2APB (100 μmol/L) + ATP (10 μmol/L) and thapsigargin (TG, 2.5 μmol/L) for 10 min in Ca^2+^-free solution. Values are shown as mean ± SEM (*n* = 3–6 experiments). ^*^P < 0.05 for scrambled siRNA vs. TRPP2 or STIM1 or TRPP2 + STIM1 siRNA transfection in each treatment. **d** Representative images showing STIM1 puncta formation in the mouse aortic smooth muscle cells transfected with TRPP2 siRNA or scrambled siRNA and treated by ATP (10 μmol/L), 2APB (100 μmol/L) + ATP (10 μmol/L) and thapsigargin (TG, 2.5 μmol/L) for 10 min in Ca^2+^-free solution. Scale bar represents 10 μm. The experiment was repeated 4 times

To identify the function of TRPP2-STIM1 complex in the homeostasis of [Ca^2+^]_i_ in VSMCs, TRPP2 and STIM1 siRNAs were used to suppress TRPP2 and STIM1 expression in the primary cultured mouse aortic VSMCs (Supplementary Fig. 2a,b). TRPP2 siRNA did not affect Orai1, STIM1 and IP_3_R expression. In addition, STIM1 siRNA did not affect Orai1, TRPP2 and IP_3_R expression (Supplementary Fig. 2c,d). The [Ca^2+^]_i_ measurement results showed that TRPP2, STIM1 or TRPP2 + STIM1 siRNA transfection strongly suppressed the ATP-induced SOCE as compared to scrambled siRNA control in the primary cultured VSMCs (Fig. 5a, c). STIM1 siRNA with or without TRPP2 siRNA transfection markedly decreased the TG-induced SOCE but TRPP2 siRNA merely moderately suppressed the TG-induced SOCE as compared to scrambled siRNA control (Fig. 5c). The transfection with TRPP2 siRNA alone or together with STIM1 siRNA significantly suppressed the ATP- and TG-induced Ca^2+^ release (Fig. 5b). Furthermore, the ATP-induced SOCE and Ca^2+^ release were abolished by 2APB (Fig. 5b).

It has been well-documented that the Ca^2+^ store depletion will evoke STIM1 puncta formation which then activates Orai1 channels. The immunofluorescent experiments demonstrated that TRPP2 siRNA dramatically suppressed ATP-induced STIM1 puncta formation in the primary cultured VSMCs (Fig. 5d). 2APB abolished the ATP-induced STIM1 puncta formation in both groups (Fig. 5d). Meanwhile, no significant difference in TG-induced STIM1 puncta formation was found between scrambled siRNA control and TRPP2 siRNA transfection (Fig. 5d). The data indicated that the TRPP2-specific regulation of the STIM1 puncta formation was IP_3_R dependent.

Therefore, these results indicate that TRPP2 and STIM1 associate together in VSMCs involving agonist-induced SOCE.

### Functional role of TRPP2-STIM1 association in agonist-induced contraction in endothelium denuded mouse aorta

The activation of G protein-coupled receptors (GPCRs) causes phospholipase Cβ to convert phosphatidylinositol 4,5-biphosphate into IP_3_ and diacylglycerol [43]. The IP_3_ activates TRPP2 indirectly via the Ca^2+^ release from IP_3_R [8]. Certain GPCRs are able to increase the [Ca^2+^]_i_ via evoking the Ca^2+^ release and SOCE to contract VSMCs. Therefore, the TRPP2-STIM1 association is potentially important in the VSMCs contraction and blood vessel tone regulation. To identify the functional role of TRPP2-STIM1 complex, the tension of mouse aorta was measured. Unfortunately, no chemicals can specifically inhibit TRPP2 or STIM1. Thus, we used organ culture to transfect TRPP2 siRNA or STIM1 siRNA into isolated mouse aorta to suppress TRPP2 or STIM1 expression [10]. Immunofluorescence showed that TRPP2 and STIM1 specific siRNAs dramatically suppressed TRPP2 and STIM1 protein expression in the cultured mouse aorta (Supplementary Fig. 3).

**Fig. 6:**
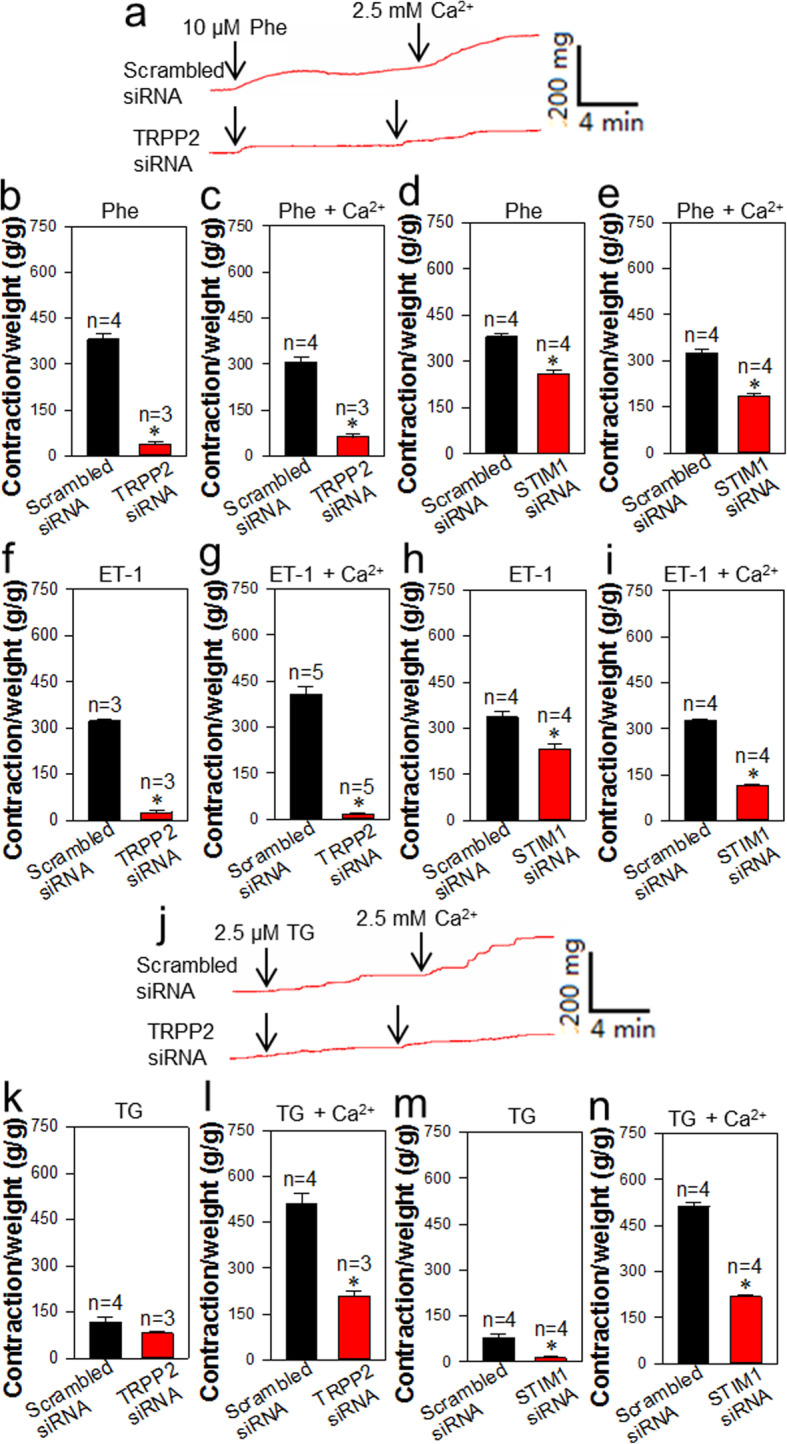
Role of TRPP2 and STIM1 in Ca^2+^ release and store-operated Ca^2+^ entry (SOCE)-induced mouse aorta contraction. **a** Representative traces showing phenylephrine (Phe, 10 μmol/L)-induced contraction in Ca^2+^-free solution and extracellular Ca^2+^ (2.5 mmol/L) re-addition-induced contraction in mice aortae. **b**-**e** Summarized data showing Phe-induced contraction in Ca^2+^-free solution (**b**, **d**) and extracellular Ca^2+^ re-addition (**c**, **e**)-induced contractions in mice aortae, which were transfected with TRPP2 siRNA (**b**, **c**), SITM1 siRNA (**d**, **e**) or scrambled siRNA. **f**-**i** Summarized data showing endothelin 1 (ET-1, 100 nmom/L)-induced contraction in Ca^2+^-free solution (**f**, **h**) and extracellular Ca^2+^ re-addition (**g**, **i**)-induced contractions in mice aortae transfected with TRPP2 siRNA (**f**, **g**), SITM1 siRNA (**h**, **i**) or scrambled siRNA. Values are shown as mean ± SEM (*n* = 3–4 mice). ^*^P < 0.05 for scrambled siRNA vs. TRPP2 siRNA or STIM1 siRNA transfection. **j** Representative traces showing thapsigargin (TG, 2.5 μmol/L)-induced contraction in Ca^2+^-free solution and extracellular Ca^2+^ (2.5 mmol/L) re-addition-induced contraction in mice aortae. **k**-**n** Summarized data showing TG-induced contraction in Ca^2+^-free solution (**k**, **m**) and extracellular Ca^2+^ re-addition (**l**, **n**)-induced contraction in mice aortae, which were transfected with TRPP2 siRNA (**j**-**l**), SITM1 siRNA (**m**-**n**) or scrambled siRNAs. Values are shown as the mean ± SEM (*n* = 3–4 mice). ^*^P < 0.05 for TRPP2 siRNA or STIM1 siRNA vs. scrambled siRNA

In the tension measurement, the endothelial layer of mouse aorta was removed for specifically investigating the VSMC contraction. The results showed that Phe (10 μmol/L), ET-1 (100 nmol/L) and TG (2.5 μmol/L) induced the vessel contraction in the Ca^2+^-free solution due to the Ca^2+^ release from the Ca^2+^ store (Fig. 6). Subsequent re-addition of 2.5 mmol/L Ca^2+^ into bath solution induced further contraction because of the SOCE (Fig. 6). Interestingly, TRPP2 siRNA markedly suppressed Phe- and ET-1-induced contraction but slightly inhibited TG-induced contraction as compared to scrambled siRNA controls in the Ca^2+^-free solution (Fig. 6a, b, f, j, k). Moreover, TRPP2 siRNA markedly suppressed SOCE-induced contraction in Phe, ET-1 and TG treatments (Fig. 6a, c, g, j, i). On the other hand, STIM1 siRNA significantly decreased the Phe-, ET-1- and TG-induced contraction in the Ca^2+^-free solution (Fig. 6d, h, m) and the SOCE-induced contraction as compared to scrambled siRNA controls (Fig. 6e, i, n).

**Fig. 7:**
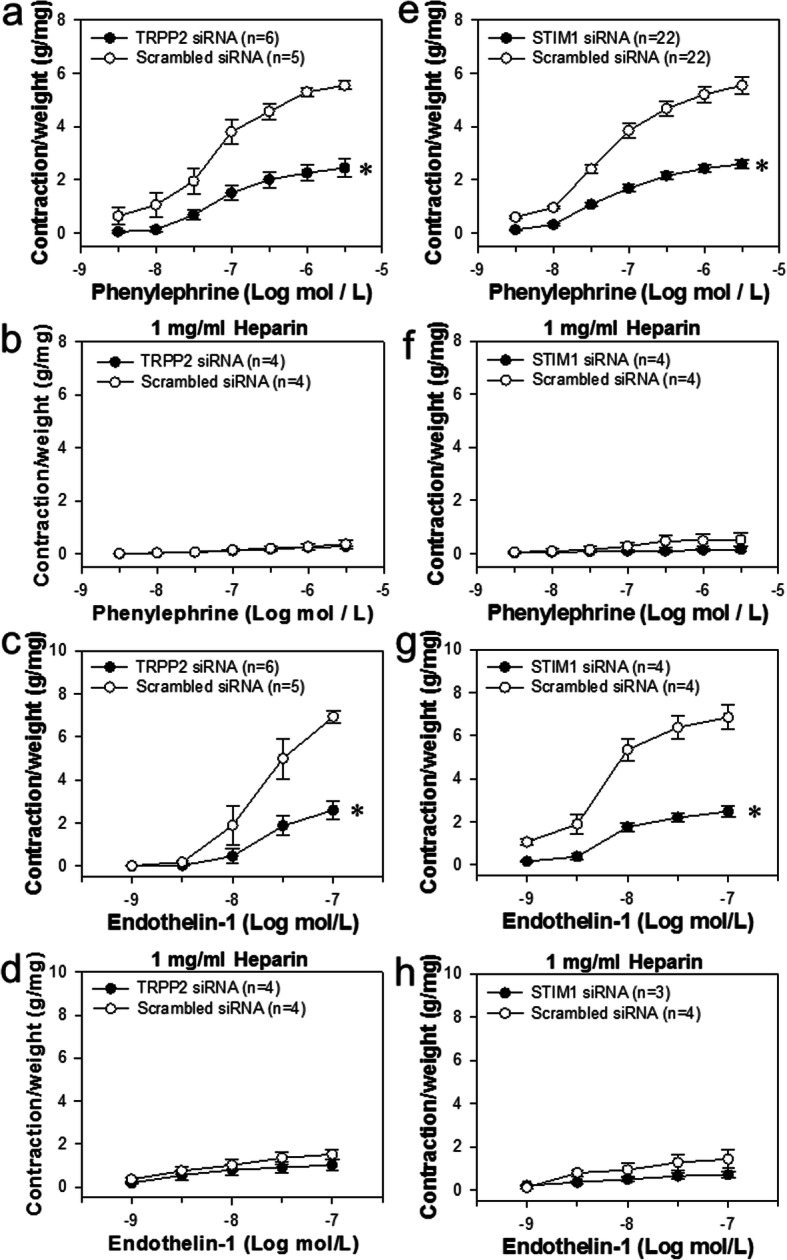
Role of TRPP2 and STIM1 in agonist-induced mouse aorta contraction. Phenylephrine (10 μmol/L, **a**, **b**, **e**, **f**) and endothelin 1 (100 nmol/L, **c**, **d**, **g**, **h**) concentration-dependently induced the contraction of the mice aortae transfected with TRPP2 (**a**-**d**), STIM1 (**e**-**h**) or scrambled siRNA. **b**, **d**, **f**, **h** The mice aortae were pretreated by heparin (1 mg/ml) using a reversible permeabilization loading procedure. Values are shown as mean ± SEM (*n* = 4–6 mice). ^*^P < 0.05 for scrambled siRNA vs. TRPP2 or STIM1 siRNA transfection

To further investigate the involvement of the TRPP2-STIM1 association in the agonist-induced vessel contraction, Phe- and ET-1-induced dose-dependent contractions between TRPP2 siRNA, STIM1 siRNA and scrambled siRNA transfection in mouse aorta were compared. The data indicated that TRPP2 or STIM1 siRNA separately suppressed the Phe- and ET-1-induced dose-dependent contraction in normal Krebs’ solution as compared to respective scrambled siRNA controls (Fig. 7a, c, e, g). More importantly, after the pretreatment of IP_3_R antagonist heparin (1 mg/mL), the differences between TRPP2 siRNA or STIM1 siRNA and respective controls were abolished (Fig. 7b, d, f, h), further supporting the notion that TRPP2 regulates the store Ca^2+^ release and SOCE necessary for the agonist-induced contraction, which needs IP_3_R activation.

**Fig. 8:**
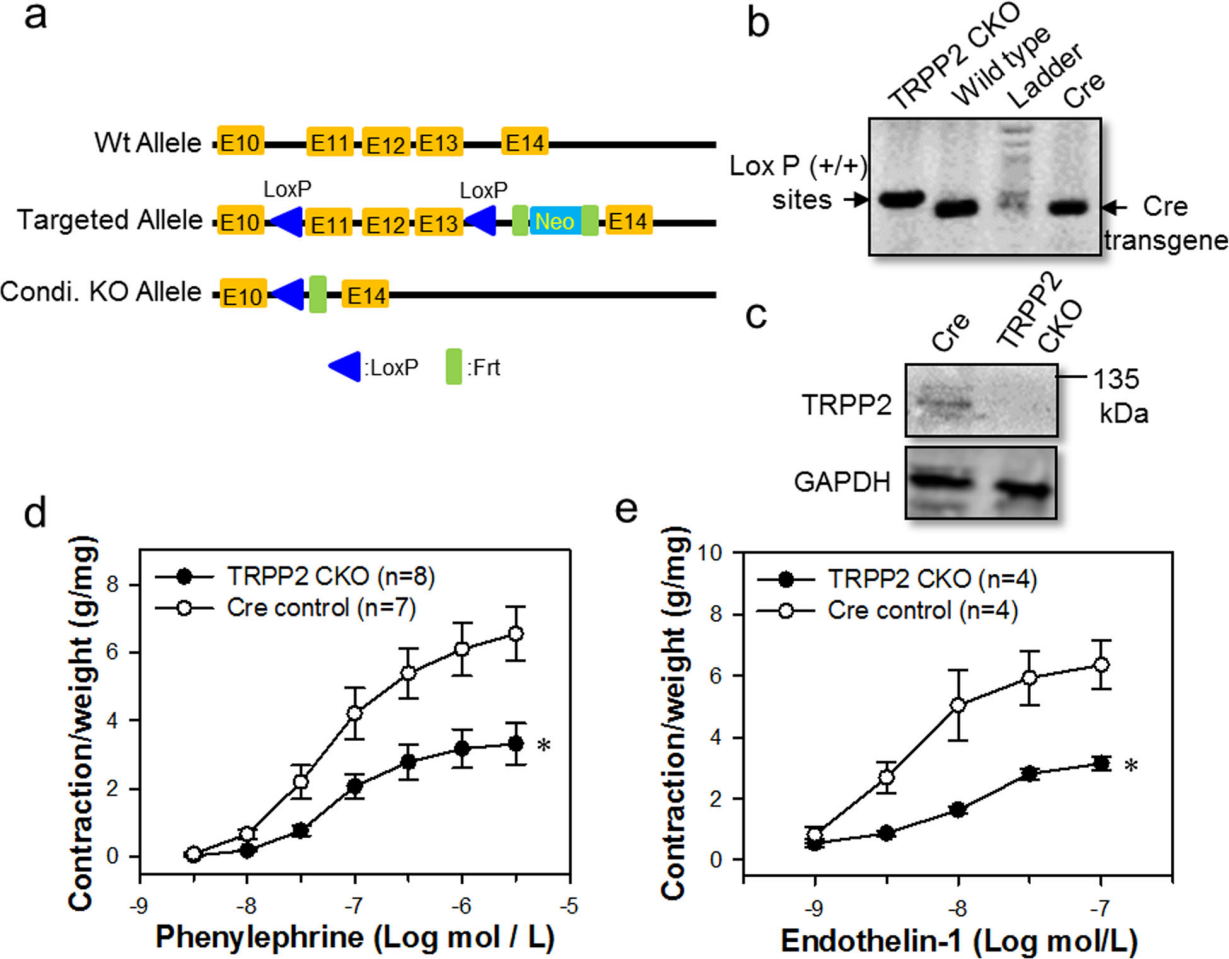
Generation and verification of the TRPP2 conditional knockout (CKO) mice. **a** Schematic description of gene-targeting map of *PKD2* gene. Targeting construct is referring to the floxed allele. Flp-recombined is obtained from deletion of the Neomycin cassette. Cre-recombined is the mutant allele yielded from the deletion with Cre-recombinase. **b** Genomic PCR analysis data from mice tail DNA samples showing the presence of LoxP^+/+^ sites and Cre-transgens. **c** Immunoblots data showing TRPP2 expressing in aortic smooth muscle cells from TRPP2 CKO or Cre control mice. **d**, **e** Agonist-induced mouse aorta contraction. Phenylephrine (10 μmol/L, **d**) and endothelin 1 (100 nmol/L, **e**) concentration-dependently induced the contraction of TRPP2 CKO or Cre control mice aortae. Values are shown as mean ± SEM (*n* = 4–8 mice). ^*^P < 0.05 for Cre control vs. TRPP2 CKO mouse

CKO mouse was a very powerful tool for investigating the functional role of TRPP2 in agonist-induced vessel contraction. To create TRPP2 CKO mouse, two *LoxP* sites were inserted between *PKD2* exon (Fig. 8a). Tagln-Cre mice were used to cross with *PKD2 LoxP* mice to specially delete *PKD2* gene in smooth muscle and heart muscle tissues. The genotyping was used to identify TRPP2 CKO mice (Fig. 8b). Immunoblots data indicated that TRPP2 protein was not expressed in TRPP2 CKO mice aortic smooth muscle cells compared to Tagln-Cre control mice (Fig. 8c). Phe- and ET-1-induced vessel contractions were significantly decreased in TRPP2 CKO mice compared to Tagln-Cre control mice (Fig. 8d, e).

Taken together, these results very strongly supported our finding that TRPP2 was a crucial component participating in agonist-induced VSMCs contraction and blood vessel tone.

## Discussion

TRPP2, is the prototypical member of TRP channel superfamily, expressed in large amounts in the ER membrane and acting as a calcium release channel [44]. Here, we demonstrated that TRPP2 associates with STIM1 to form a signaling complex to regulate SOCE, VSMCs contraction and blood vessel tone, enriching our understanding of this channel and its role in blood vessel contraction.

Accumulating evidence suggests that molecules in the same signaling pathway often form a complex for effective signal transduction. TRPP2 mainly serves a Ca^2+^ release channel in the ER membrane, while STIM1 N-terminus which contains the Ca^2+^ sensing domain also locates in the ER lumen. The data of the present study strongly suggest that TRPP2 and STIM1 associate each other to form a signal complex. In our Ca^2+^ microdomain model: (1) the opening of TRPP2 will quickly decrease local Ca^2+^ levels near the channel pore region within the ER lumen; (2) this reduction in local Ca^2+^ level is sensed by nearby N-terminus of STIM1. Through this association, the local Ca^2+^ concentration reduction in the ER lumen will activate STIM1 with high efficiency and sensitivity. Santoso et al. reported that TRPP2 competes with STIM1 to associate with IP_3_R and also found that TRPP2 did not pull down STIM1 in Madin-Darby Canine Kidney (MDCK) epithelial cells [45]. However, our results unambiguously showed that TRPP2 and STIM1 pulled down each other both in TRPP2-STIM1 co-expressing HEK293 cells and native VSMCs. It is possible that the interaction of TRPP2 and STIM1 is cell type-dependent.

Qian et al. reported that *Pkd*2^+/−^ mouse displayed a reduced TRPP2 expression and decreased SOCE in VSMCs [46]. ATP is able to act on the purinergic membrane receptors to produce IP_3_ and then activate TRPP2 [8]. Here, we used TRPP2 siRNA to knockdown TRPP2 protein expression in the primary cultured VSMCs and mouse aortic VSMCs. The Ca^2+^ measurement data showed that suppressing TRPP2 expression significantly decreased the ATP- and TG-induced Ca^2+^ release and SOCE. IP_3_R antagonist 2APB abolished the ATP-induced Ca^2+^ release and SOCE. The results demonstrate that suppressing TRPP2 expression decrease the SOCE, which is consistent with Qian’s report [46]. As we expected, STIM1 siRNA did not affect the ATP- and TG-induced Ca^2+^ release but markedly reduced the ATP- and TG-induced SOCE. The ATP-induced STIM1 puncta formation was also obviously decreased by TRPP2 siRNA. These data strongly suggest that TRPP2-STIM1 complex participates in agonist-induced [Ca^2+^]_i_ increase. In addition, the vessel tension measurement showed that TRPP2 siRNA and STIM1 siRNAs dramatically decreased the Phe- and ET-1-induced contraction in the Ca^2+^-free solution and the SOCE-induced contraction. TRPP2 siRNA slightly decreased the TG-induced contraction in the Ca^2+^-free solution and significantly suppressed the SOCE-induced contraction. STIM1 siRNA significantly decreased the TG-induced contraction in the Ca^2+^-free solution and the SOCE-induced contraction. It is well known that Phe and ET-1 act on GPCR to produce IP_3_ which leads to Ca^2+^ release from IP_3_R. TG can induce the Ca^2+^ release via IP_3_R-dependent and independent pathways [38]. TRPP2 is then activated by the Ca^2+^ release from IP_3_R to mediate further Ca^2+^ release from the ER [8]. Therefore, these vessel tension measurement data demonstrate that TRPP2-STIM1 complex is crucially responsible for the agonist-induced vessel contraction via the Ca^2+^ release and SOCE.

In a dose-dependent response of mouse aorta, TRPP2 siRNA and STIM1 siRNA strongly suppressed the Phe- and ET-1-induced vessel contraction, which is consistent with the result of the contraction evoked by the Phe- and ET-1-induced Ca^2+^ release and SOCE. Moreover, heparin treatment using a reversible permeabilization loading procedure to inhibit IP_3_R abolished the Phe- and ET-1-induced contraction. More importantly, the Phe- and ET-1-induced contractions were significantly reduced in TRPP2 CKO mouse aorta. The data indicate that IP_3_R-mediated Ca^2+^ release is an initial factor for the agonist-induced vessel contraction and TRPP2-STIM1 complex may be the downstream of this signal pathway. Taken together, we speculate that TRPP2-STIM1 complex may importantly participate in the agonist-induced Ca^2+^ release, SOCE and contraction in VSMCs.

There are two more issues worth discussing. (1) How does TRPP2 affect the Ca^2+^ release from the ER? Qian’s report and our study support that TRPP2 knockdown decreased the Ca^2+^ release from the ER [46]. On the contrary, Wegierski et al. reported that TRPP2 knockdown augmented the ER Ca^2+^ release in MDCK cells [12]. These controversies could result from the experimental design or cell background. (2) How does TRPP2 involve in the VSMC contraction? We used TRPP2-specific siRNA to suppress TRPP2 expression. Our data demonstrate that TRPP2 knockdown significantly decreased the agonist-induced contraction of mouse aorta. But, Qian et al. found that the Phe-induced contraction of mouse aorta was increased in *Pkd*2^+/−^ mouse [47]. Note that, Qian et al. suggested that the Phe-induced contraction was Ca^2+^-independent [47] and they attributed the increased contraction to an elevated expressions in smooth muscle α-actin and myosin heavy chain in *Pkd*2^+/−^ arteries [47]. More importantly, in the present study when TRPP2 was conditionally knocked out in smooth muscle cells, the Phe- and ET-1-evoked contractions both were markedly reduced. We speculate that the discrepancy in the results regarding the Phe-induced aortic contraction between us and Qian’s could be due to an altered protein expression in *Pkd*2^+/−^ mouse.

## Conclusion

In summary, we demonstrate that TRPP2 physically associates with STIM1 to form a signal complex in VSMCs, and that this Ca^2+^ microdomain formed by TRPP2-STIM1 complex plays an important functional role in the agonist-induced Ca^2+^ release, SOCE and vessel contraction.

## Supplementary information

**Additional file 1.**

## Data Availability

Datasets and non-commercial materials can be obtained from the corresponding author on reasonable request.

## Abbreviations

TRPP2, PKD2: Polycystin-2
STIM1: Stormal interaction molecule 1
SOCE: Store-operated Ca^2+^ entry
VSMCs: Vascular smooth muscle cells
Phe: Phenylephrine
ET-1: Endothelial 1
2APB: 2-aminoethyl diphenylborinate
ER: Endoplasmic reticulum
TG: Thapsigargin
PLA: Proximity ligation assay
FRET: Fluorescence resonance energy transfer
GPCRs: G protein-coupled receptors
IP_3_R: 1,4,5-triphosphate receptor
CKO: Conditional knockout
