# Supplementary material for: TRPP2 and STIM1 form a microdomain to regulate store-operated Ca2+ entry and blood vessel tone
Source: Cell Commun Signal. 2020 Aug 31;18:138. doi: 10.1186/s12964-020-00560-7 (PMC7457527; doi:10.1186/s12964-020-00560-7)
Supplement: Supplementary file 2 — Additional file 1. [file 12964_2020_560_MOESM2_ESM.pdf]

Supplementary Figure 1

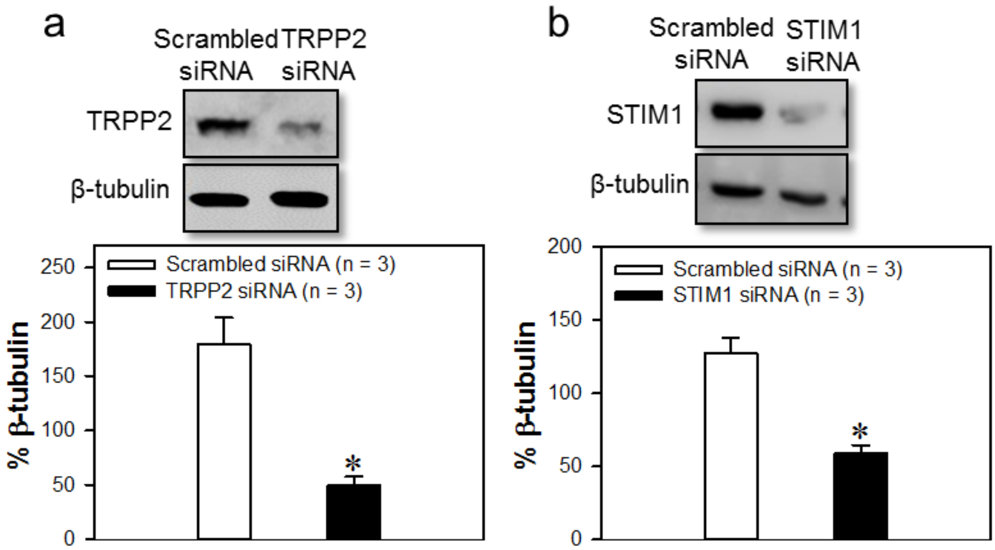

Supplementary Figure 1. Effects of TRPP2 siRNA and STIM1 siRNAs on TRPP2 and STIM1 protein expression levels in HEK293 cells. (**a**) Representative images (upper) and summarized data (lower) of TRPP2 siRNA effect on TRPP2 protein expression in HEK293 cells. (**b**) Representative images (upper) and summarized data (lower) of STIM1 siRNA effect on STIM1 protein expression in HEK293 cells. Values are shown as the mean ± SEM (n = 3). *$P < 0.05$ for TRPP2 or STIM1 siRNA *vs.* scrambled siRNA.

Supplementary Figure 2

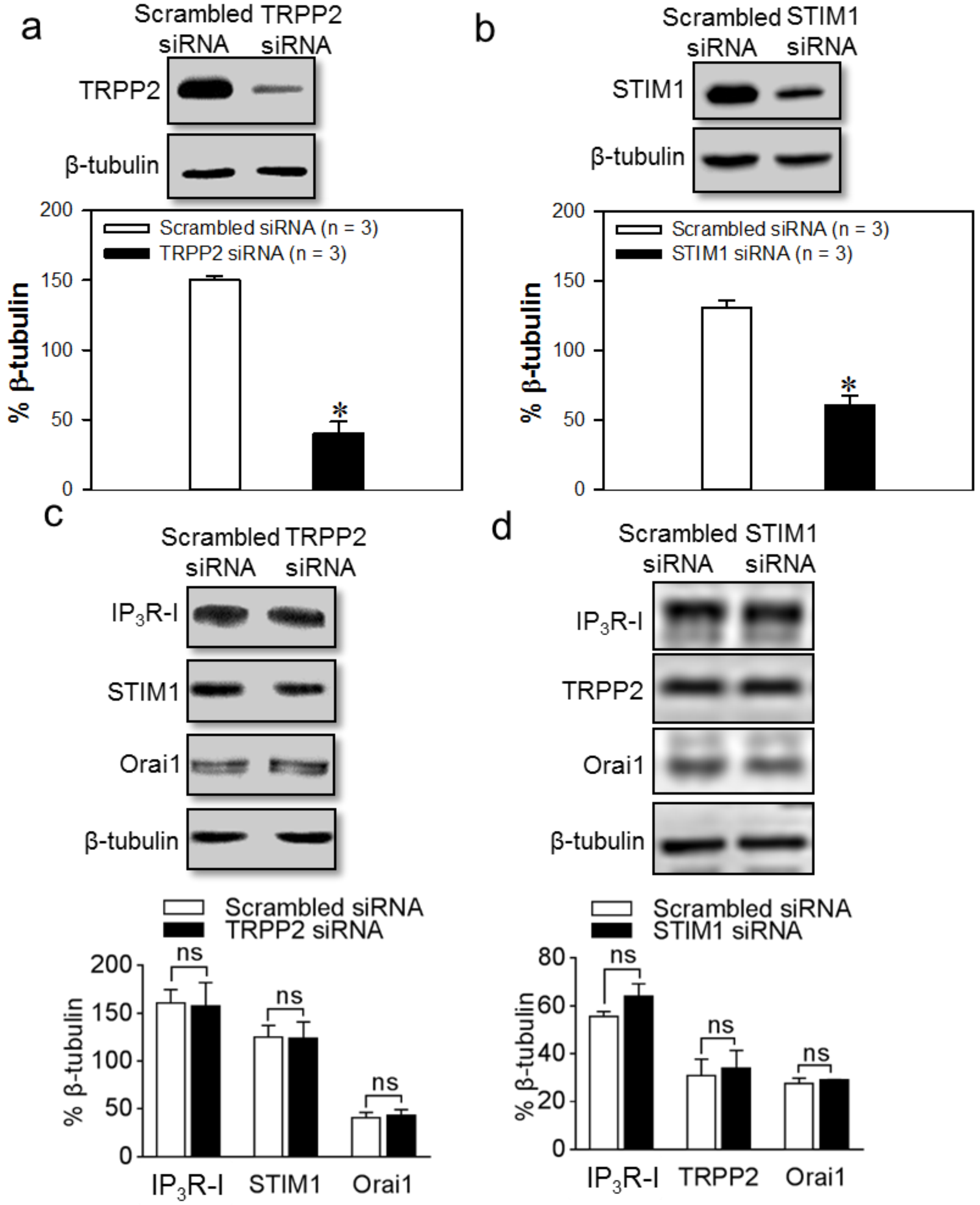

Supplementary Figure 2. Effects of TRPP2 siRNA and STIM1 siRNAs on TRPP2, STIM1, Orai1 and IP_3R-I protein expression levels in primary cultured vascular smooth muscle cells (VSMCs). (**a**) Representative images (upper) and summarized data (lower) of TRPP2 siRNA effect on TRPP2 protein expression in VSMCs. (**b**) Representative images (upper) and summarized data (lower) of STIM1 siRNA effect on STIM1 protein expression in VSMCs. Protein expressions were normalized to β-tubulin. Values are shown as the mean ± SEM (n = 3). *$P < 0.05$ for TRPP2 siRNA or STIM1 siRNA vs. scrambled siRNA. (**c**) Representative images (upper) and summarized data (lower) of TRPP2 siRNA effect on IP_3R-I, STIM1 and Orai1 protein expression levels in VSMCs. (**d**) Representative images (upper) and summarized data (lower) of STIM1 siRNA effect on IP_3R-I, TRPP2 and Orai1 protein expression levels in VSMCs. Protein expressions were normalized to β-tubulin. Values are shown as the mean ± SEM (n = 3).

Supplementary Figure 3

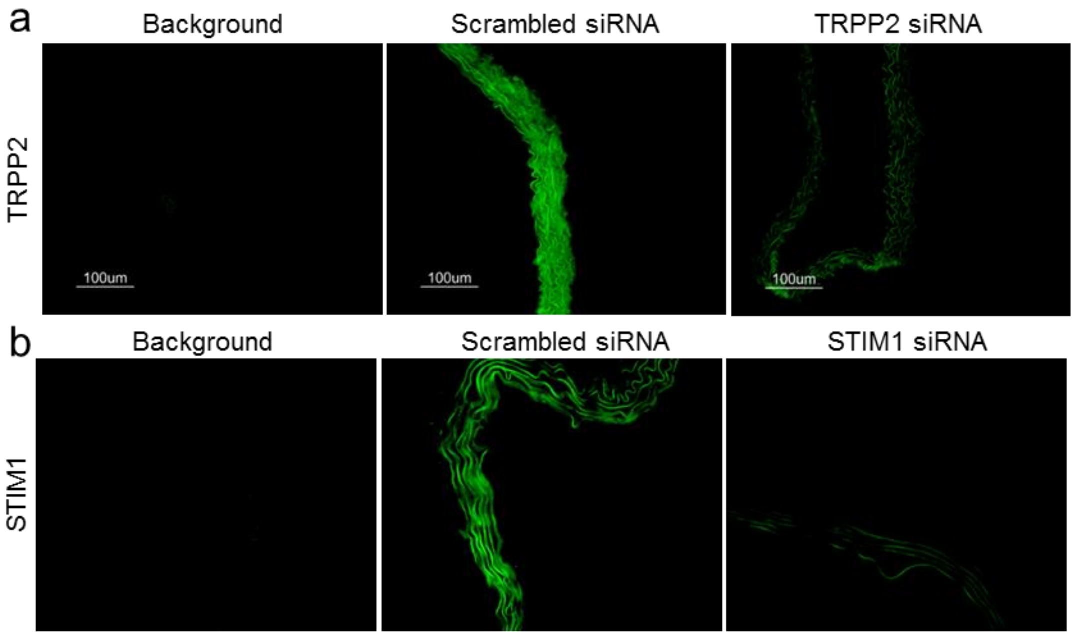

Supplementary Figure 3. Effects of TRPP2 siRNA and STIM1 siRNAs on TRPP2 and STIM1 protein expression levels in mice aortae. (**a**,**b**) Representative immunofuorescence images showing TRPP2 (**a**) and STIM1 (**b**) protein expression in the mice aortae with or without TRPP2 or STIM1 siRNA treatment. Background images were no primary antibody incubation.
